# 

**Published:** 1896-12

**Authors:** 


### INDEX TO VOLUME XVII.

A.

Abbott, Frank, M.D. On Dental Pathology and Practice, 397.
Academy of Stomatology, 163, 314, 517.
A Case of Calcification of a Widely Exposed Pulp, 503.
Acute Dentinal Sensibility, Electrical Osmosis in the Treatment of, 341.
Address of Dr. A. V. Elliott, of Florence, Italy, Presidential, 640.
Adenoid Vegetation, Nasal Obstructions which result in Mouth-Breathing, with Special References to, 549.
After-thoughts, 390.
A Hint to Dental Students, 332.
A History of the Chronic Degenerative Diseases of the Central Nervous System, 328.
A Journalistic War, 395.
Alveolaris, Pyorrhœa, 203, 717.
American Academy of Dental Science, 94, 310, 376, 446, 508, 737, 806.
American Dental Association, 23, 153, 220, 475, 659, 720, 788.
American Dental Society of Europe, 403.
American Medical Association, 337.
Anæsthesia, Dental, by Cataphoresis, 151.
Anæsthesia, Formula for Local, 684.
Anæsthetic, Eucaine Hydrochlorate, a New Local, 572.
An Apology to the Editor of the *Digest*, 252.
An Apparent Case of Salivation, 561.
A New Hæmostatic, 340.
A New Local Anæsthetic, Eucaine Hydrochlorate, 572.
A New Stand-Point, Pyorrhœa Alveolaris from, 774.
An Important Correction, 330.
An Interesting Case, 370.
Anniversary, The Golden, 334.
Annual of the Universal Medical Sciences, 62.
An Old Subject revived, 542.
An Oral Camera for Röntgen Photography, 497.
Antipyretics, The Value of, 764.
Antiseptic, Loretin, the New, 441.
Antrum of Highmore, Empyema of the, 405.
Applications of Cataphoresis, The Principles and, 418.
A Röntgen Ray Converter for Internal Use, 445.
Association of Faculties, Dr. Williams and the, 545.
A Substitute for Iodoform, Salubrol, 566.
A System of Detachable Facings, 488.
A Talk on Materia Medica, 294.
A True History of the Discovery of Anæsthesia. A Reply to Mrs. E. Whitman Morton, 834.

B.

Bacteria of the Mouth, Some Points in Connection with the, 642.
Beaten on Bridge Suit, Crown Company, 253.
Bennett, Mr. Storer. On Report of a Case of Reunited Fractured Human Tooth, 718.
Bethel, L. P., D.D.S. On Lining Root-Canals, 723.
Bibliography: A History of the Chronic Degenerative Diseases of the Central Nervous System, 328.
- Annual of the Universal Medical Sciences, 62.
- A True History of the Discovery of Anæsthesia. A Reply to Mrs. E. Whitman Morton, 834.
- Catching's Compendium of Practical Dentistry for 1895, 255.
- Dental Chemistry and Metallurgy, 254.
- Dental Pathology and Practice, 397.
- Electricity in Electro-Therapeutics, 396.
- Extraction of the Teeth, 680.
- Kronen und Brücken Arbeiten, 61.
- Lehrbuch der Conservirenden Zahnheilkunde, 395.
- Notes on the History of Anæsthesia, etc., 833.
- Pathologies des Dents et de la Bouche, 327.
- Practical Dental Metallurgy, 832.
- The American Text-Book of Prosthetic Dentistry in Contributions by Eminent Authors, 756.
- The Diseases of Children's Teeth: Their Prevention and Treatment, 126.
- The Principles and Practice of Dentistry, including Anatomy, etc., 476.
- Transactions of the American Dental Association at the Thirty-fifth Annual Session, held at Asbury Park, N. J., 1895, 680.

Breneman, Professor A. A. On Some of the Physical Properties of Metals, 355.

Bridge Suit, Crown Company beaten on, 253.
Briggs, Dr. E. C. On a Talk on Materia Medica, 294.
Brown, G. Carleton, D.D.S. On Dental Examining Boards in the United States and Preliminary Education, 87.
Brown, Peter, L D.S. On Suggestions on the Use of External Osmosis in Dental Practice, 197.
On the Advantages of Cataphoresis in Dental Operations, 365.

C.

Calcification of a Widely Exposed Pulp, A Case of, 503.
Calculi, Salivary, 621.
Case, An Interesting, 370.
Cataphoresis, 704.
Cataphoresis, Dental Anæsthesia by, 151.
Cataphoresis in Dental Operations, The Advantages of, 365.
Cataphoresis, Skeleton Notes on, 783.
Cataphoresis, The Principles and Applications of, 418.
Catching, B. H., D.D.S. On Catching's Compendium on Practical Dentistry for 1895, 255.
Central Dental Association of Northern New Jersey, 527, 599.
Central Nervous System, A History of the Chronic Degenerative Diseases of the, 328.
Certain Manifestations of Syphilis of Importance in the Practice of Dentistry, 685.
Chandler, Dr. Thomas H. Resolutions of Respect to the Memory of, 63, 128.
Chemistry, Metallurgy and Dental, 254.
Chicago Dental Society, 402.
Children's Teeth, The Diseases of: Their Prevention and Treatment, 126.
Childs, Dr. E. L., 835.
Chronic Degenerative Diseases of the Central Nervous System, A History of the, 328.
Cincinnati Academy of Dentistry, 480.
Clark, Wm. E., S.B. On Electricity and its Applications in Dentistry, 694.
Clinical Considerations on Infectious Gingivitis, 481.
Cobb, Frederic C., M.D. On Empyema of the Antrum of Highmore, 405.
Cocaine, 431.
Cocaine, Fatal Acute Poisoning by, 683.
Colton, G. Q. A True History of the Discovery of Anæsthesia, 834.
Colyer, J. F. On Extraction of the Teeth, 680.
Compendium of Practical Dentistry for 1895, Catching's, 255.
Constitutional Defect, Dental and Facial Evidences of, 261.
Corrected, The History of Cataphoresis, 678.
Correction, An Important, 330.
Criticism, Dr. Williams's, 192.
Crown Company beaten on Bridge Suit, 253.
Cumston, Charles Greene, B.M.S., M.D. On Clinical Considerations on Infectious Gingivitis, 481.
Current News: American Dental Society of Europe, 403.
American Medical Association, 337.
Chicago Dental Society, 402.
Cincinnati Academy of Dentistry, 480.
Dental Society of the State of New York, 338.
Harvard Dental Alumnia Association, 548.
Illinois State Dental Society, 479.
Interstate Dental Meeting, 404.
Louisiana State Dental Society, 338.
Meeting in Western Pennsylvania, 64.
National Association of Dental Examiners, 479.
New Jersey State Dental Society, 479, 836.
New Orleans Academy of Stomatology, 339.
North Carolina State Dental Society, 403.
Northeastern Dental Association, 258, 683, 835.
Odontographic Society of Chicago, 132.
Pennsylvania State Dental Examining Board, 402.
Pennsylvania State Dental Society, 402.
Philadelphia County Dental Society, 763.
The Fifty-first Anniversary of Horace Wells's Discovery, 403.
The Golden Anniversary, 334.
Topics for Discussion by State and Local Organizations, 132.
Woman's Dental Association, 196, 259.
Custer, Levitt E., B.S., D.D.S. On Electric Heat in Dental Practice, 1.

D.

Darby, Dr. George D. B. On Is Uric Acid an Important Factor in Dental Disease? 143.
Davis, Philip W., A.B., S.B. On Electricity and its Applications in Dentistry, 694.
December Thoughts, 828.
Defect, Dental and Facial Evidences of Constitutional, 261.
Delayed Matter, 327.
Detachable Facings, a System of, 488.
Dental Anæsthesia by Cataphoresis, 151.
Dental and Facial Evidences of Constitutional Defect, 261.
Dental Chemistry and Metallurgy, 254.
Dental Colleges, The War on the, 122.
Dental Disease, Is Uric Acid an Important Factor in, 143.
Dental Examining Boards in the United States and Preliminary Education, 87.
Dental Operations, The Advantages of Cataphoresis in, 365.

Dental Pathology and Practice, 397.
Dental Practice, Electric Heat in, 1.
Dental Practice, Suggestions on the Use of Electrical Osmosis in, 197.
Dental Practitioner, The Qualifications Necessary to the, 500.
Dental Society of the State of New York, 338.
Dental Uses for Röntgen's Discovery.—Support for Tube, 559.
Dentinal Sensibility, Electrical Osmosis in the Treatment of Acute, 341.
Dentistry electrically considered, The Future of, 491.
Dentistry, Electricity and its Applications to, 694.
Dentistry, Practical and Theoretical, 563.
Dentistry, Progressive, 17.
De Terra, Paul. Salubrol a Substitute for Iodoform, 566.
Directly Connected Static Machines, 635.
Disinfectants, 339.
Domestic Correspondence: An Important Correction, 330.
  Louisville Odontological Society, 330.
  The History of Cataphoresis corrected, 678.
Dr. C. W. Spalding, 547.
Dr. Edwin A. Stebbins, 762, 834.
Dr. E. L. Childs, 835.
Dr. G. V. Black's Investigation of the Human Teeth, 57.
Dr. Phineas G. C. Hunt, 399.
Dr. W. E. Magill, 478.
Dr. Williams and the Association of Faculties, 545.
Dr. Williams's Criticism, 192.
Dr. Williams's Retort, 753.
Dubois, Paul, 400.
Duplication and Reduplication of Papers, 250.
Dwinelle, Dr. W. H., 193, 256, 328, 400.

E.

Editorial: After-Thoughts, 390.
  A Journalistic War, 395.
  An Apology to the Editor of the *Digest*. 252.
  An Old Subject Revived, 542.
  Crown Company beaten on Bridge Suit, 253.
  December Thoughts, 828.
  Delayed Matter, 327.
  Dr. G. V. Black's Investigations of the Human Teeth, 57.
  Dr. Williams and the Association of Faculties, 545.
  Dr. Williams's Criticism, 192.
  Dr. Williams's Retort, 753.
  Dr. W. H. Dwinelle, 193.
  Duplication and Reduplication of Papers, 250.
  Electrical Osmosis, 393.
  Examining Boards and their Selection, 749.
  Explanation, 126.
Editorial:
  Is Reorganization Necessary? 674.
  Personal Explanation, 327.
  Saratoga, 1896, 617.
  Solila Gold, 394.
  The American Dental Association, 475.
  The Art of Professional Teaching, 324.
  The Parasite in Science, 190.
  The Separation of the National Associations, 830.
  The Vacation Period, 472.
  The War on the Dental Colleges, 122.
Editorial, Reply to the January, 194.
Editor of the *Digest*, An Apology to the, 252.
Electric Heat in Dental Practice, 1.
Electrical Osmosis, 393.
Electrical Osmosis for the Treatment of Living Dentine, 65.
Electrical Osmosis in the Treatment of Acute Dentinal Sensibility, 341.
Electrical Osmosis, Suggestions on the Use of, in Dental Practice, 197.
Electricity and its Applications in Dentistry, 694.
Electricity in Electro-Therapeutics, 396.
Electro-Therapeutics, Electricity in, 396.
Empyema of the Antrum of Highmore, 405.
Essig, Chas. J., M.D., D.D.S. The American Text-Book of Prosthetic Dentistry in Contributions by Eminent Authorities, 756.
Eucaine Hydrochlorate, 576.
Eucaine Hydrochlorate, a New Local Anæsthetic, 572.
Examining Boards and their Selection, 749.
Explanation, 126.
Explanation, Personal, 327.
Exposed Pulp, A Case of Calcification of a Widely, 503.
Extraction of the Teeth, 680.

F.

Facings, A System of Detachable, 488.
Faculties, Dr. Williams and the Association of, 545.
Fatal Acute Poisoning by Cocaine, 683.
Fillings, Porcelain, 713.
Finishing Cement Fillings, 332.
Förberg, Elof. On Porcelain Fillings, 713.
Foreign Correspondence: Editorial, Reply to the January, 194.
  Reply to the January Editorial, 194.
Formic Aldehyde, Notes on, 286.
Formula for Local Anæsthesia, 684.
Frey, Dr. Léon. On Pathologie des Dents et de la Bouche, 327.
Frisk, Theo., D.D.S. On Solila, 372.
Function of the Vacuum-Chamber, 682.
Fütterer, Gustav, M.D. On Salivary Calculi, 620.

G.

Garretson, Professor James E., In Memory of, 131.
Gilbert, S. Eldred, D.D.S. On Loretin, the New Antiseptic, 441.

Gillett, Henry W., M.D. On Cataphoresis, 704.
On Electrical Osmosis for the Treatment of Living Dentine, 65.
Gingivitis, Clinical Considerations on Infectious, 481.
Goadby, K. W. On Some Points in Connection with Bacteria of the Mouth, 642.
Goldsmith, Dr. S. L. On an Interesting Case, 370.
Gold Solder Scraps, The Use of, 331.
Grant, Dr. George F. On Hypnotism: Its Value to the Dental Specialist, 83.
Guy, G. H. On the Future of Dentistry electrically considered, 491.

H.

Hæmostatic, A New, 340.
Harris, Chapin A., M.D., D.D.S. On Principles and Practice of Dentistry, including Anatomy, etc., 476.
Harvard Dental Alumni Association, 548.
Harvard Odontological Society, 41, 260.
Head, Joseph, M.D., D.D.S. On the Application of the Principle of Crown-Work to Metal Plates, 637.
Headridge, P., L.D.S.I. On Putting a Porcelain Facing on a Living Honey-Combed Incisor, 787.
Heat, Electric, in Dental Practice, 1.
Hodgen, Joseph Dupuy, D.D.S. Practical Dental Metallurgy, 832.
Honey-Combed Incisor, Putting a Porcelain Facing on a Living, 787.
Hopkins, Samuel A., M.D. On Cocaine, 434.
Houston, Edwin J. On Electricity in Electro-Therapeutics, 396.
Howe, J. Morgan, M.D., M.D.S. On Notes on Formic Aldehyde, 286.
Hughes, Rev. Edwin H. On the Profession and the Man, 9.
Human Teeth, Dr. G. V. Black's Investigations of the, 57.
Hunt, Dr. Phineas G. C., 399.
Hydrochlorate, Eucaine, 576.
Hypnotism: Its Value to the Dental Specialist, 83.

I.

Illinois State Dental Society, 479.
Infectious Gingivitis, Clinical Considerations on, 481.
In Memory of Professor James E. Garretson, 131.
Interesting Case, An, 370.
Internal Use, A Röntgen Ray Converter for, 445.
Inter-State Dental Meeting, 404.
Investigations of the Human Teeth, Dr. G. V. Black's, 57.
Iodoform, Salubrol a Substitute for, 566.
Is Reorganization Necessary? 674.
Is Uric Acid an Important Factor in Dental Disease? 143.

J.

Jack, Louis, D.D.S. On Electrical Osmosis in the Treatment of Acute Dentinal Sensibility, 341.
On Plantation of Teeth, 133.
Skeleton Notes on Cataphoresis, 783.

K.

Kennelly, A. E. On Electricity in Electro-Therapeutics, 396.
Kiesel, H. On Eucaine Hydrochlorate, 576.
Kronen und Brücken Arbeiten, 61.

L.

Laceration in Extraction, Remedy for, 480.
Lehrbuch der Conservirenden Zahnheilkunde, 395.
"Light," On, 345.
Lighting the Mouth, 371.
Lining Root-Canals, 723.
Living Dentine, Electrical Osmosis for the Treatment of, 65.
Local Anæsthesia, Formula for, 684.
Loretin, the New Antiseptic, 441.
Louisiana State Dental Society, 338.
Louisville Odontological Society, 330.

M.

Magill, Dr. W. E., 478.
Man, The Profession and the, 9.
Mason, W. L., D.D.S. On a System of Detachable Facings, 488.
Materia Medica, A Talk on, 294.
Matter, Delayed, 327.
McManus, James, D.D.S. Notes on the History of Anæsthesia, etc., 833.
Meeting in Western Pennsylvania, 64.
Metal Plates, The Application of the Principles of Crown-Work to, 637.
Metals, Some of the Physical Properties of, 355.
Metallurgy, Dental Chemistry and, 254.
Miller, von W. D. Lehrbuch der Conservirenden Zahnheilkunde, 395.
Mills, Dr. G. A. On "Resistibility," 94.
On What can we demonstrate? 639.
Mitchell, Clifford, A.M., M.D. On Dental Chemistry and Metallurgy, 254.
Monroe, T. Kirkpatrick, M.A., M.D. On a History of the Chronic Degenerative Diseases of the Central Nervous System, 328.
Mouth-Breathing, Nasal Obstructions which Result in Special Reference to Adenoid Vegetations, 549.
Mouth, Lighting the, 371.

N.

Nasal Obstructions which result in Mouth-Breathing, with Special Reference to Adenoid Vegetations, 549.
National Association of Dental Examiners, 479.

National Association of Dental Faculties, 652.
National Associations, The Separation of the, 830.
New Formula for Local Anæsthetic, 682.
New Jersey State Dental Society, 479, 836.
New Orleans Academy of Stomatology, 339.
New York Odontological Society, 175.
North Carolina State Dental Society, 403.
Northeastern Dental Association, 258, 683, 835.
NOTES AND COMMENTS: A Hint to Dental Students, 332.
Finishing Cement Fillings, 332.
Function of the Vacuum-Chamber, 682.
New Formula for Local Anæsthetic, 682.
Notes on Formic Aldehyde, 286.
Pluck, not Luck, 681.
Pyorrhœa Alveolaris, 682.
Religion of the Body, 333.
Removal of Blood Spots, 332.
The Dentist's Position, 331.
The Open Mind, 333.
The Use of Gold Solder Scraps, 331.
Trichloracetic Acid in cleaning Teeth, 332.
We may learn from the Humblest, 681.
Notes on the History of Anæsthesia. The Wells Memorial Celebration, etc., 833.

O.

OBITUARY: Chandler, Dr. Thomas H., Resolutions of Respect to the Memory of, 63, 128.
Childs, Dr. E. L., 835.
Dubois, Paul, 400.
Dwinelle, William H., A.M., M.D., D.D.S., 193, 256, 328, 400.
Garretson, Professor James E., In Memory of, 232.
Hunt, Dr. Phineas G. C., 399.
Magill, Dr. W. E., 478.
Spalding, Dr. C. W., 547.
Stebbins, Dr. Edwin A., 762.
Resolution of Respect, 834.
Tucker, Elisha G., M.D., 131.
White, John De Haven, M.D., D.D.S., 62, 129.
Odontographic Society of Chicago, 132.
Odontological Society of Pennsylvania, 52, 667, 747.
On "Light," 345.
Operations, The Advantages of Cataphoresis in Dental, 365.
Oral Electricity and Vital Currents, 215.
ORIGINAL COMMUNICATIONS: An Apparent Case of Salivation, 561.
An Interesting Case, 370.
An Oral Camera for Röntgen Photography, 497.
A Röntgen Ray Converter for Internal Use, 445.
A System of Detachable Facings, 488.
ORIGINAL COMMUNICATIONS:
A Talk on Materia Medica, 294.
Cataphoresis, 704.
Certain Manifestations of Syphilis of Importance in the Practice of Dentistry, 685.
Clinical Considerations on Infectious Gingivitis, 481.
Cocaine, 431.
Dental Anæsthesia by Cataphoresis, 151.
Dental and Facial Evidences of Constitutional Defect, 261.
Dental Examining Boards in the United States and Preliminary Education, 87.
Dental Uses for Röntgen's Discovery. —Support for Tube, 559.
Dentistry, Practical and Theoretical, 563.
Directly Connected Static Machines, 635.
Electric Heat in Dental Practice, 1.
Electrical Osmosis for the Treatment of Living Dentine, 65.
Electrical Osmosis in the Treatment of Acute Dentinal Sensibility, 341.
Electricity and its Applications in Dentistry, 694.
Empyema of the Antrum of Highmore, 405.
Hypnotism: Its Value to the Dental Specialist, 83.
Is Uric Acid an Important Factor in Dental Disease? 143.
Lighting the Mouth, 371.
Loretin, the New Antiseptic, 441.
Nasal Obstructions which result in Mouth-Breathing, with Reference to Adenoid Vegetations, 549.
Notes on Formic Aldehyde, 286.
On "Light," 345.
Oral Electricity and Vital Currents, 215.
Plantation of Teeth, 133.
Porcelain Fillings, 713.
Presidential Address of Dr. A. V. Elliott, Florence, Italy, 640.
Progressive Dentistry, 17.
Pyorrhœa Alveolaris, 203, 717.
Pyorrhœa Alveolaris from a New Stand-Point, 774.
"Resistibility," 94.
Salivary Calculi, 621.
Skeleton Notes on Cataphoresis, 783.
Some of the Physical Properties of Metals, 355.
Suggestions on the Use of Electrical Osmosis in Dental Practice, 197.
The Advantages of Cataphoresis in Dental Operations, 365.
The Application of the Principle of Crown-Work to Metal Plates, 637.
The Future of Dentistry electrically considered, 491.
The Principles and Applications of Cataphoresis, 418.
The Profession and the Man, 9.

Original Communications:
The Qualifications Necessary for the Dental Practitioner, 500.
The Röntgen Ray, 765.
Third Set of Teeth, 371.
What can we demonstrate? 639.
Osmosis, Electrical, 393.

P.

Palmer, S. B., M.D.S., on Oral Electricity and Vital Currents, 215.
Papers, Duplication and Reduplication of, 250.
Parachlorophenol, 339.
Pathologie des Dents et de la Bouche, 327.
Pathology and Practice, Dental, 397.
Paul Dubois, 400.
Pennsylvania State Dental Examining Board, 402.
Pennsylvania State Dental Society, 402.
Period, The Vacation, 473.
Personal Explanation, 327.
Philadelphia County Dental Society, 763.
Phillips, Wendell C., M.D., on the Principles and Applications of Cataphoresis, 418.
Photography, An Oral Camera for Röntgen, 497.
Physical Properties of Metals, Some of the, 355.
Plantation of Teeth, 133.
Pluck, not Luck, 681.
Poisoning by Cocaine, Fatal, Acute, 683.
Porcelain Fillings, 713.
Position, The Dentist's, 331.
Practical and Theoretical Dentistry, 563.
Practical Dental Metallurgy, 832.
Practical Dentistry for 1895, Catching's Compendium on, 255.
Practice, Dental Pathology and, 397.
Practice, Electric Heat in Dental, 1.
Practitioner, The Qualifications Necessary for the Dental, 500.
Preliminary Education, Dental Examining Boards in the United States and, 87.
Presidential Address of Dr. A. V. Elliott, Florence, Italy.
Prevention and Treatment of Diseases of Children's Teeth, 126.
Principles and Applications of Cataphoresis, The, 418.
Professional Teaching, The Art of, 324.
Progressive Dentistry, 17.
Properties of Metals, Some of the Physical, 35.
Putting a Porcelain Facing on a Living Honey-Combed Incisor, 787.
Pyorrhœa Alveolaris, 203, 682, 717.
Pyorrhœ Alveolaris, from a New Stand-Point, 774.

Q.

Qualifications Necessary for the Dental Practitioner, 500.

R.

Reduplication of Papers, Duplication and, 250.
Religion of the Body, 333.
Remedy for Laceration in Extraction, 480.
Removal of Blood-Spots, 332.
Reply to the January Editorial, 194.
Report of Society Meetings: Academy of Stomatology, 163, 314, 517, 821.
American Academy of Dental Science, 94, 310, 376, 446, 508, 737, 806.
American Dental Association, 23, 153, 220, 475, 659, 720, 788.
Central Dental Association of Northern New Jersey, 527, 599.
Harvard Odontological Society, 41, 260.
Lining Root-Canals, 723.
National Association of Dental Faculties, 652.
New York Odontological Society, 175.
Odontological Society of Pennsylvania, 52, 667, 747.
The New York Institute of Stomatology, 105, 235, 300, 385, 461, 580.
Report on a Case of Reunited Fractured Human Tooth, 718.
"Resistibility," 94.
Resolution of Respect to the Memory of Dr. E. A. Stebbins, 834.
Resolutions of Respect to the Memory of Dr. Thomas H. Chandler, 63, 128.
Reunited Fractured Human Tooth, Report on a Case of, 718.
Revived, An Old Subject, 542.
Richards, George L., M.D. Nasal Obstructions which Result in Mouth-Breathing, with Special Reference to Adenoid Vegetations, 549.
Rollins, William. On an Oral Camera for Röntgen Photography, 497.
On a Röntgen Ray Converter for Internal Use, 445.
On Dental Anæsthesia by Cataphoresis, 151.
On Dental Uses for Röntgen's Discovery.—Support for Tube, 559.
On Directly-Connected Static Machines, 635.
On Lighting the Mouth, 371.
Röntgen's Discovery, Dental Uses for.—Support for Tube, 559.
Röntgen Photography, An Oral Camera for, 497.
Root-Canals, Lining, 723.

S.

Sajous, Charles E., M.D., Annual of the Universal Medical Sciences, 62.
Salivary Calculi, 621.
Salivation, An Apparent Case of, 561.
Salubrol, A Substitute for Iodoform, 566.
Saratoga, 1896, 617.
Science, The Parasite in, 190.
Scraps, The Use of Gold Solder, 331.

SELECTIONS: A New Hæmostatic, 340.
Disinfectants, 339.
Fatal Acute Poisoning by Cocaine, 683.
Formula for Local Anæsthesia, 684.
Parachlorophenol, 339.
Remedy for Laceration in Extraction, 480.
The Medical Profession in the United States, 684.
The Value of Antipyretics, 764.
Sensibility, Electrical Osmosis in the Treatment of Acute Dentinal, 341.
Skeleton Notes on Cataphoresis, 783.
Smith, Dr. P. T., on the Qualifications Necessary for the Dental Practitioner, 500.
Smith, William Lincoln. On The Röntgen Ray, 765.
Solila, 372.
Solila Gold, 394.
Some of the Physical Properties of Metals, 355.
Some Points in Connection with the Bacteria of the Mouth, 642.
Spalding, Dr. C. W., 547.
Specialist, Hypnotism, Its Value to the Dental, 83.
Stainton, C. W., D.D.S. On an Apparent Case of Salivation, 561.
Standish, Myles, M.D. On "Light," 345.
State and Local Organizations, Topics for Discussion by, 132.
Static Machines, Directly Connected, 635.
Stebbins, Dr. Edwin A., 762.
Subject Revived, An Old, 542.
Suggestions on the Use of Electrical Osmosis in Dental Practice, 197.
Support for Tube. Dental Uses for Röntgen's Discovery, 559.
Syphilis of Importance in the Practice of Dentistry, Certain Manifestations of, 685.

T.

Talbot, Eugene S., M.D., D.D.S. On Dental and Facial Evidences of Constitutional Defect, 261.
On Pyorrhœa Alveolaris, 203, 717.
Teaching, The Art of Professional, 324.
Teeth, Plantation of, 133.
Teeth, Third Set of, 371.
The Advantages of Cataphoresis in Dental Operations, 365.
The American Text-Book of Prosthetic Dentistry in Contributions by Eminent Authors, 756.
The Application of the Principle of Crown-Work to Metal Plates, 637.
The Art of Professional Teaching, 324.
The Dentist's Position, 331.
The *Digest*, An Apology to the Editor of, 252.
The Diseases of Children's Teeth: Their Prevention and Treatment, 126.
The Fifty-first Anniversary of Horace Wells's Discovery, 403.
The Future of Dentistry Electrically Considered, 491.
The Golden Anniversary, 334.
The History of Cataphoresis corrected, 678.
The Medical Profession in the United States, 684.
The Mouth, Lighting, 371.
The New Antiseptic, Loretin, 441.
The New York Institute of Stomatology, 105, 235, 300, 385, 461, 581.
The Open Mind, 333.
Theoretical Dentistry, Practical and, 563.
The Parasites in Science, 190.
The Principles and Applications of Cataphoresis, 418.
The Principles and Practice of Dentistry, including Anatomy, etc., 476.
The Profession and the Man, 9.
The Qualifications Necessary for the Dental Pratitioner, 500.
The Röntgen Ray, 765.
The Separation of the National Associations, 830.
The Use of Gold Solder Scraps, 331.
The Vacation Period, 472.
The Value of Antipyretics, 764.
The War on the Dental Colleges, 122.
Third Set of Teeth, 371.
Thoughts, After-, 390.
Thoughts, December, 828.
Tomes, Charles S. A Case of Calcification of a Widely Exposed Pulp, 503.
Topics for Discussion by State and Local Organizations, 132.
To the Editor of the *Digest*, An Apology, 252.
Townsend, J. C., D.D.S., on Progessive Dentistry, 17.
Transactions of the American Dental Association at the Thirty-fifth Annual Session, held at Asbury Park, N. J., 1895, 680.
TRANSLATIONS: A Case of Calcification of a Widely Exposed Pulp, 503.
Eucaine Hydrochlorate, 576.
Eucaine Hydrochlorate, A New Local Anæsthetic, 572.
Putting a Porcelain Facing on a Living Honey-Combed Incisor, 787.
Report on a Case of Reunited Fractured Human Tooth, 718.
Salubrol, a Substitute for Iodoform, 566.
Solila, 372.
Some Points in connection with the Bacteria of the Mouth, 642.
Treatment of Acute Dentinal Sensibility, Electrical Osmosis in the, 341.
Treatment of Living Dentine, Electrical Osmosis for the, 65.
Trichloracetic Acid in Cleaning Teeth, 332.
Trueman, Wm. H., D.D.S. On Pyorrhœa Alveolaris from a New Stand-Point, 774.
Tucker, Elisha G., M.D., 131.
Twilley, William Shaw. On Dentistry, Practical and Theoretical, 563.

U.

Use of Electrical Osmosis in Dental Practice, Sugestions on the, 197.

V.

Vacation Period, The, 472.
Vinci, Dr. Gaetano. Eucaine Hydrochlorate, a New Local Anæsthetic, 572.
Vital Currents, Oral Electricity and, 215.

W.

War, A Journalistic, 395.
Washbourn, J. W. On Some Points in Connection with the Bacteria of the Mouth, 642.
We may learn from the Humblest, 681.
What can we demonstrate? 639.
White, John DeHaven, M.D., D.D.S., 63, 129.
Whitney, C. M., M.D. Certain Manifestations of Syphilis of Importance in the Practice of Dentistry, 685.
Woman's Dental Association, 196, 259.

SUBSCRIBE FOR THE

### International Dental Journal,

EDITED BY JAMES TRUMAN, D.D.S.

### Published by the International Dental Publication Company.

LOUIS JACK, PRESIDENT, Philadelphia.
BENJAMIN LORD, VICE-PRESIDENT, New York.
W. H. TRUEMAN, TREASURER, Philadelphia.
GEO. S. ALLAN, SECRETARY,
51 West Thirty-seventh St., New York.

**Publication Office, 716 Filbert St., Philadelphia, Pa.**

### STOCKHOLDERS.

George Emory Adams . . South Orange, N.J.
Geo. S. Allan . . . . . . . . New York, N.Y.
R. R. Andrews . . . . . . . . . Cambridge, Mass.
H. A. Baker . . . . . . . . . . . Boston, Mass.
A. E. Baldwin . . . . . . . . . Chicago, Ill.
W. C. Barrett . . . . . . . . . . Buffalo, N.Y.
*A. P. Beale . . . . . . . . . Philadelphia, Pa.
S. T. Beale, Jr. . . . . . . . . Philadelphia, Pa.
C. S. Beck . . . . . . . . . . . Wilkesbarre, Pa.
G. V. Black . . . . . . . . . Jacksonville, Fla.
C. F. W. Bodecker . . . . . . New York, N.Y.
E. A. Bogue . . . . . . . . . . New York, N.Y.
W. G. A. Bonwill . . . . . Philadelphia, Pa.
C. A. Brackett . . . . . . . . . Newport, R. I.
Thomas Bradley . . . . . . New York, N.Y.
Edward C. Briggs . . . . . . . . Boston, Mass.
Albert H. Brockway . . . . . Brooklyn, N.Y.
A. E. Brown . . . . . . . . . . . Chicago, Ill.
Wm. Carr . . . . . . . . . . New York, N.Y.
Geo. H. Chance . . . . . . Portland, Oregon.
G. F. Cheney . . . . . . . . St. Johnsbury, Vt.
Dwight M. Clapp . . . . . . . . Boston, Mass.
John T. Codman . . . . . . . . . Boston, Mass.
Chas. D. Cook . . . . . . . . . Brooklyn, N.Y.
J. N. Crouse . . . . . . . . . . . Chicago, Ill.
Edw. T. Darby . . . . . . . . Philadelphia, Pa.
H. S. Draper . . . . . . . . . . . Boston, Mass.
Wm. H. Dwinelle . . . . . . New York, N.Y.
A. R. Eaton . . . . . . . . . . . Elizabeth, N.J.
Albert T. Emery . . . . . . . . Boston, Mass.
Chas. J. Essig . . . . . . . . . Philadelphia, Pa.
J. N. Farrer . . . . . . . . . New York, N.Y.
T. Fillebrown . . . . . . . . . . Portland, Me.
W. B. Finney . . . . . . . . . . Baltimore, Md.
T. A. Fletcher . . . . . . . . New York, N.Y.
M. W. Foster . . . . . . . . . . Baltimore, Md.
Chas. E. Francis . . . . . . . New York, N.Y.
E. C. Fundenberg . . . . . . . . Pittsburg, Pa.
W. F. Fundenberg . . . . . . . . Pittsburg, Pa.
W. H. Fundenberg . . . . . . . . Pittsburg, Pa.
E. S. Gaylord . . . . . . . New Haven, Conn.
J. P. Geran . . . . . . . . . . . Brooklyn, N.Y.
A. H. Gilson . . . . . . . . . . . Boston, Mass.
S. H. Guilford . . . . . . . . Philadelphia, Pa.
John I. Hart . . . . . . . . New York, N.Y.
O. E. Hill . . . . . . . . . . . Brooklyn, N.Y.
J. F. P. Hodson . . . . . . . . New York, N.Y.
J. Morgan Howe . . . . . . . New York, N.Y.
Robert Huey . . . . . . . . Philadelphia, Pa.
A. O. Hunt . . . . . . . . . . Iowa City, Iowa.
Louis Jack . . . . . . . . . Philadelphia, Pa.
V. H. Jackson . . . . . . . . New York, N.Y.
C. R. Jefferis . . . . . . . . Wilmington, Del.
*C. A. Kingsbury . . . . . . Philadelphia, Pa.
W T. La Roche . . . . Harrington Park, N.J.
W. K. Leech . . . . . . . . . . Philadelphia, Pa.
*Fred. A. Levy . . . . . . . . . . Orange, N.J.
Wilbur F. Litch . . . . . . . Philadelphia, Pa.
J. Bond Littig . . . . . . . . . New York, N.Y.
Benjamin Lord . . . . . . . New York, N.Y.
Geo. W. Lovejoy . . . . . . . . . Montreal, Ont.
John S. Marshall . . . . . . . . . Chicago, Ill.
H. J. McKellops . . . . . . . . . St. Louis, Mo.
Charles McManus . . . . . Hartford, Conn.
James McManus . . . . . . . . Hartford, Conn.
Dan'l Neal McQuillan . . . Philadelphia, Pa.
Frederic A. Merrill . . . . . . Boston, Mass.
Horatio C. Meriam . . . . . . . . Salem, Mass.
W. D. Miller . . . . . . . . . . Berlin, Germany.
Geo. T. Moffatt . . . . . . . . . Boston, Mass.
A. L. Northrop . . . . . . . New York, N.Y.
W. E. Page . . . . . . . . . . . . Boston, Mass.
S. B. Palmer . . . . . . . . . . . Syracuse, N.Y.
C. B. Parker . . . . . . . . . . . Brooklyn, N.Y.
D. D. Peabody . . . . . . . . Stoneham, Mass.
C. N. Peirce . . . . . . . . . . Philadelphia, Pa.
S. G. Perry . . . . . . . . . . New York, N.Y.
W. A. Phreaner . . . . . . . Philadelphia, Pa.
Chas. E. Pike . . . . . . . . Philadelphia, Pa.
W. E. Pinkham . . . . . . . . . New York, N.Y.
W. H. Potter . . . . . . . . . . . . Boston, Mass.
Chas. P. Pruyn . . . . . . . . . . . Chicago, Ill.
H. C. Register . . . . . . . . . Philadelphia, Pa.
F. A. Roy . . . . . . . . . . . . New Orleans, La.
Z. T. Sailer . . . . . . . . . . New York, N.Y.
L. D. Shepard . . . . . . . . . . . Boston, Mass.
Geo. R. Shidle . . . . . . . . . . Pittsburg, Pa.
Eugene H. Smith . . . . . . . . . Boston, Mass.
B. Holly Smith . . . . . . . . . Baltimore, Md.
H. A. Smith . . . . . . . . . . . Cincinnati, Ohio.
S. G. Stevens . . . . . . . . . . . . Boston, Mass.
W. X. Sudduth . . . . . . Minneapolis, Minn.
Eugene S. Talbott . . . . . . . Chicago, Ill.
*Ambler Tees . . . . . . . . . Philadelphia, Pa.
J. D. Thomas . . . . . . . . . Philadelphia, Pa.
James Truman . . . . . . . . Philadelphia, Pa.
Wm. H. Trueman . . . . . . . Philadelphia, Pa.
W. W. Walker . . . . . . . . . New York, N.Y.
I. F. Wardwell . . . . . . . . . Stanford, Conn.
G. W. Warren . . . . . . . . . Philadelphia, Pa.
T. S. Waters . . . . . . . . . . . Baltimore, Md.
Geo. W. Weld . . . . . . . . New York, N.Y.
Julius G. W. Werner . . . . . . Boston, Mass.
Jacob L. Williams . . . . . . . . Boston, Mass.
*R. B. Winder . . . . . . . . . Baltimore, Md.
C. A. Woodward . . . . . . . . New York, N.Y.
J. A. Woodward . . . . . . . Philadelphia, Pa.
W. A. Woodward . . . . . . New York, N.Y.
W. J. Younger . . . . . . . San Francisco, Cal.

**Deceased.*

The officers of the International Dental Publication Company serve without salary, the investments of the stockholders merely guarantee the success of the enterprise, ALL PROFITS being devoted to the enlargement or improvement of the International Dental Journal.

**The support of every dentist having the welfare of his profession at heart is earnestly solicited. Subscription, $2.50 per Annum.**

*THE INTERNATIONAL DENTAL PUBLICATION CO.*

